# Artesunate Ameliorates SLE Atherosclerosis Through PPARγ-Driven Cholesterol Efflux Restoration and Disruption of Lipid Raft-Organized TLR9/MyD88 Signaling Pathway

**DOI:** 10.3390/biom15081078

**Published:** 2025-07-25

**Authors:** Miao Zhang, Xinyu Pan, Yuanfang He, Kairong Sun, Zhiyu Wang, Weiyu Tian, Haonan Qiu, Yiqi Wang, Chengping Wen, Juan Chen

**Affiliations:** 1 Institute of Basic Research in Clinical Medicine, College of Basic Medical Science, Zhejiang Chinese Medical University, Hangzhou 310000, China; 202311010211015@zcmu.edu.cn (M.Z.); 202311115911077@zcmu.edu.cn (X.P.); 202411115911018@zcmu.edu.cn (K.S.); 2 Key Laboratory of Chinese Medicine Rheumatology of Zhejiang Province, Hangzhou 310000, China; 3 Zhejiang Provincial Zhongshan Hospital, The Third Affiliated Hospital of Zhejiang Chinese Medical University, Hangzhou 310000, China; 4 The Second Clinical Medical College, Zhejiang Chinese Medical University, Hangzhou 310000, China; 202212210909010@zcmu.edu.cn; 5 Ningbo Taikang Hospital, Ningbo 315000, China; anny228123@163.com; 6 The Children’s Hospital, Zhejiang University School of Medicine, National Clinical Research Center for Child Health, Hangzhou 310029, China; haonanqiu06@gmail.com; 7 School of Pharmaceutical Science, Zhejiang Chinese Medical University, Hangzhou 310000, China; wangyiqi@zcmu.edu.cn

**Keywords:** SLE atherosclerosis, cholesterol efflux, cholesterol homeostasis, inflammation, lipid raft, TLR9/MyD88

## Abstract

Systemic lupus erythematosus (SLE) is characterized by autoimmune dysregulation, elevated autoantibody production, and persistent inflammation, predisposing patients to atherosclerosis (AS). Atherogenesis is dependent on lipid homeostasis and inflammatory processes, with the formation of lipid-laden, macrophage-derived foam cells (MDFC) essential for atherosclerotic lesion progression. Elevated cholesterol levels within lipid rafts trigger heightened pro-inflammatory responses in macrophages via Toll-like receptor 9 (TLR9). Artesunate (ART), an artemisinin derivative sourced from *Artemisia annua*, exhibits therapeutic potential in modulating inflammation and autoimmune conditions. Nonetheless, its impact and mechanisms in SLE-associated AS (SLE-AS) remain largely unexplored. Our investigation demonstrated that ART could effectively ameliorate lupus-like symptoms and atherosclerotic plaque development in SLE-AS mice. Moreover, ART enhanced cholesterol efflux from MDFC by upregulating ABCA1, ABCG1, and SR-B1 both in vivo and in vitro. Moreover, ART reduced cholesterol accumulation in bone marrow-derived macrophages (BMDMs), thereby diminishing TLR9 recruitment to lipid rafts. ART also suppressed TLR9 expression and its downstream effectors in the kidney and aorta of SLE-AS mice, attenuating the TLR9-mediated inflammatory cascade in CPG2395 (ODN2395)-stimulated macrophages. Through bioinformatics analysis and experimental validation, PPARγ was identified as a pivotal downstream mediator of ART in macrophages. Depleting PPARγ levels reduced the expression of ABCA1, ABCG1, and SR-B1 in macrophages, consequently impeding cholesterol efflux. In conclusion, these findings suggest that ART ameliorates SLE-AS by restoring cholesterol homeostasis through the PPARγ-ABCA1/ABCG1/SR-B1 pathway and suppressing lipid raft-driven TLR9/MyD88 inflammation.

## 1. Introduction

Systemic lupus erythematosus (SLE) is a chronic autoimmune disease characterized by the production of autoantibodies, deposition of immune complexes, and systemic inflammation, which may affect multiple organs [1,2]. The precise pathogenesis and diagnostic criteria for systemic lupus erythematosus remain to be elucidated. Atherosclerosis (AS) is a progressive vascular disease driven by lipid accumulation, endothelial dysfunction, and chronic inflammation [3], leading to atherosclerotic plaque formation. Recent research suggests that SLE patients exhibit a significantly higher risk of developing premature atherosclerosis [4], a condition termed SLE-associated atherosclerosis (SLE-AS). The pathogenesis of SLE-AS involves a complex interplay between immune dysregulation, chronic inflammation, and metabolic disturbances. In SLE, persistent immune activation and the release of pro-inflammatory cytokines, such as interferon-alpha (IFN-α) and interleukin-6 (IL-6), contribute to endothelial injury and promote a pro-atherogenic environment [5]. Moreover, dyslipidemia, characterized by elevated levels of oxidized low-density lipoprotein (ox-LDL) and reduced high-density lipoprotein (HDL) function, is commonly observed in SLE patients [6]. These lipid abnormalities facilitate the formation of macrophage-derived foam cells (MDFCs), a hallmark of early atherosclerotic lesions. Furthermore, the activation of pattern recognition receptors, such as Toll-like receptor 9 (TLR9), by endogenous ligands in SLE exacerbates inflammation and accelerates atherosclerosis progression [7,8]. The shared mechanisms of chronic inflammation and lipid metabolism dysregulation highlight the intricate link between SLE and atherosclerosis, underscoring the need for targeted therapies that address both immune and metabolic pathways in SLE-AS.

Peroxisome proliferator-activated receptor gamma (PPARγ), a nuclear receptor, is essential for regulating lipid metabolism [9], inflammation, and glucose homeostasis. PPARγ activation is essential for maintaining cholesterol homeostasis by promoting cholesterol efflux from macrophages [10], which is a key step in preventing foam cell formation and the development of atherosclerosis. PPARγ achieves this by upregulating the expression of ATP-binding cassette transporters A1 and G1 (ABCA1 and ABCG1) and scavenger receptor class B type I (SR-B1), which are integral membrane proteins responsible for mediating the efflux of cholesterol [11]. Dysregulation of PPARγ signaling is associated with increased intracellular cholesterol accumulation, MDFC formation, and the progression of atherosclerosis [12]. Therefore, targeting the PPARγ represents a promising therapeutic strategy for enhancing cholesterol efflux and mitigating SLE-AS-related pathologies.

Currently, glucocorticoids and immunosuppressants are used to treat SLE, and while these drugs exhibit some efficacy, long-term use can lead to hypertension, dyslipidemia, and other complications. Therefore, treating SLE-associated disease remains a clinical challenge. Artesunate (ART), a semi-synthetic artemisinin derivative of sesquiterpene lactone, is famous for its anti-malarial properties [13]. Beyond its use in treating malaria, ART has also been explored for its potential in treating diabetes, central nervous system diseases, respiratory diseases, autoimmune diseases, etc. [14,15,16,17]. It is well established that ART can significantly reduce the pro-inflammatory cytokine levels and alleviate lupus symptoms [18]. ART has also been widely used in treating inflammatory diseases, such as ischemic brain injury and asthma [19,20]. Our previous research discovered that the Qinghao decoction could alleviate renal injury and reduce aortic plaque deposition in lupus associated with atherosclerosis [21,22]. Since ART is the active ingredient of Qing Hao, and based on previous studies indicating that ART suppresses the immune system and reduces inflammation, the present research investigated ART’s impact on both lupus and atherosclerosis and explored its potential therapeutic mechanisms.

In this research, we assessed ART’s pharmacological characteristics in SLE-AS animal models, oxLDL-induced foam cell models, and CPG-2395-induced inflammation models. We explored molecular mechanisms through network pharmacology, in vivo, and in vitro experimental validation. Overall, our findings suggest that ART may offer potential therapeutic benefits in the context of SLE-AS therapy.

## 2. Materials and Methods

### 2.1. Animals

Six-week-old female *C57BL/6* mice and apolipoprotein E knockout (ApoE^-/-^) mice were supplied by Beijing Weitongda Biotechnology Co., Ltd. (Beijing, China). A Western diet (0.25% cholesterol and 20% fat) was obtained from Xietong Bioengineering (Nanjing, China). ART (Sigma-Aldrich, Merck, St. Louis, MO, USA) was dissolved in phosphate-buffered saline (PBS). All mice were randomly assigned to five groups and given an adapted diet for two weeks. In the ApoE^-/-^ group, ApoE^-/-^ mice were injected with physiological saline via intraperitoneal injection. In the model group, ApoE^-/-^ mice were injected with pristane (0.2 mL) via intraperitoneal (i.p.) injection. In the hydroxychloroquine (HCQ) group, ApoE^-/-^ mice received both pristane (i.p. injection with 0.2 mL) and an HCQ tablet (40 mg/kg). In the low-dose ART group (ART-L), ApoE^-/-^ mice received both pristane (i.p. injection) and ART (50 mg/kg). Finally, in the high-dose ART group (ART-H), ApoE^-/-^ mice received both pristane (i.p. injection) and ART (100 mg/kg). After four weeks of pristane injection, HCQ and ART were given, respectively, by gavage for 12 weeks every day. All mice were housed in specific pathogen-free (SPF) conditions and received sufficient food and water under controlled conditions (temperature 50–60%) during a 12-h light/dark cycle. Euthanasia was performed via controlled CO_2_ inhalation at a displacement rate of 30% chamber volume per minute, with cervical dislocation applied as a secondary method to ensure death. All research procedures followed the Chinese guidelines for the Care and Use of Laboratory Animals, which were approved by the Experimental Animal Management and Ethics Committee of Zhejiang Chinese Medical University, with the approval number IACUC-20200518-09.

### 2.2. Urine Protein Analysis

The urine protein levels were measured at week 4 and week 12 after drug treatments. The collected urine was centrifuged and analyzed with a quantitative detection kit (Jiancheng Bioengineering Institute, Jiangsu, China) for urine protein.

### 2.3. Enzyme-Linked Immunosorbent Assay

Autoantibody analysis: The concentrations of anti-dsDNA (CUSABIO, Wuhan, China) and antinuclear antibody (ANA) antibodies were assessed using the manufacturer’s instructions. Results are expressed in mg/mL or U/mL according to the reference standard curve.

Inflammatory cytokine analysis: The levels of IL-6, TNF-α, and IL-1β in the culture supernatants of RAW264.7 cells and kidney homogenate were quantified using ELISA kits (MultiSciences, Hangzhou, China).

### 2.4. Renal Histology Assay

The kidney tissue samples were fixed for over 24 h in 4% paraformaldehyde. Then, they were dehydrated using a graded series of alcohol concentrations in an automated dehydrator, followed by immersion in wax and embedding. The trimmed paraffin blocks were placed on a freezing table, cooled to −20 °C, and then sectioned at a thickness of 4 µm. Hematoxylin and eosin (H&E) were used to stain dewaxed paraffin sections. After that, the stained portions were dehydrated and sealed before being examined under a microscope.

### 2.5. Atherosclerotic Lesions

The mice’s hearts were removed along with the aortic roots immersed in paraformaldehyde for at least 24 h. The tissue was removed from the fixed solution and placed in sucrose solution for dehydration. Tissues were embedded with OCT (Servicebio, Wuhan, China), and serial sections were preserved at a thickness of 10 µm. Sections of the aorta’s sinus were stained with oil red O. The severity of atherosclerosis was assessed by calculating the percentage of the stained area within the total lesion area using Image J software (version 1.8.0).

### 2.6. Cell Culture

#### 2.6.1. Mouse Macrophage Cell Line

RAW 264.7 cells (Guangzhou Cellcook Biotech Co., Ltd., Guangzhou, China) cultured in 10% Fetal Bovine Serum (FBS, Gibco, Grand Island, NY, USA) and 1% Penicillin–Streptomycin solution supplemented Dulbecco’s modified Eagle’s medium (DMEM, Gibco, Grand Island, NY, USA) were maintained at 37 °C in a humidified atmosphere with 5% CO_2_. Cells were treated with ART (Sigma-Aldrich, Merck, St. Louis, MO, USA; 2.5 µM) or oligodeoxynucleotide (ODN) 2088 (tlrl-2088, Invivogen, San Diego, CA, USA; 1 µM) and then activated with ODN2395 (tlrl-2395, Invivogen, San Diego, CA, USA, American; 2 µM) to develop the inflammation model. RAW 264.7 cells were treated with 40 μg/mL ox-LDL (Solarbio, Beijing, China) for 24 h, in the presence or absence of ART.

#### 2.6.2. Bone Marrow-Derived Macrophages

The femurs and tibiae of 4-week-old ApoE^-/-^ mice were isolated under sterile conditions one month following intraperitoneal injection of pristane. Cells were obtained by flushing the bone marrow with medium and filtering through a strainer. The separated cells were cultured in IMDM (Genom, Jiaxing, China) medium enhanced with 10 ng/mL M-CSF (PRP100503, Abbkine, Wuhan, China) for seven days. These cells were pretreated with ART (2.5 µM) and the lipid raft inhibitor methyl-β-cyclodextrin (MβCD) (C4555-5G, Sigma-Aldrich, Merck, St. Louis, MO, USA), then stimulated for 24 h with 40 μg/mL of ox-LDL to develop the inflammatory model.

### 2.7. Transfection of Small Interfering RNAs

PPARγ siRNA (forward: 5′-AGUUUGCUGUGAAGUUCAAUG-3′, reverse: 5′-UUGAACUUCACAGCAAACUCA-3′) and its negative control (forward: 5′-UUCUCCGAACGUGUCACGUTT-3′, reverse: 5′-ACGUGACACGUUCGGAGAATT-3′) were transfected into cells utilizing Lipofectamine 3000 (Invivogen, San Diego, CA, USA) according to the manufacturer’s instructions. Following transfection and 48 h of cell culture, gene silencing efficiency was assessed using qRT-PCR and Western blotting.

### 2.8. Cell Counting Kit 8 (CCK8) Assay

RAW264.7 cells were seeded in 96-well plates at 10^5^ cells/well overnight before being exposed to various doses (0, 1.25, 2.5, 5, 10, and 20 μM) of ART for 24 h. Moreover, 10 μL of CCK8 (Beyotime, Shanghai, China) was added at 37 °C for 0.5 h. The CCK8 kit was then used to measure cell viability. The mean light absorption value of the six wells was calculated for each group.

### 2.9. Immunohistochemistry (IHC) Analysis

Mouse aortic tissue was sectioned (10 µm in thickness, frozen) after 4% paraformaldehyde fixation. Sections were blocked with 3% BSA for 30 min and incubated with specific primary antibodies overnight. After three washes, the slices were exposed to the secondary antibody for 50 min. Next, staining was visualized using diaminobenzidine (DBA). After counterstaining the nuclei, dehydration, and sealing the slices, images were examined and captured using a microscope (ONIVA). The following antibodies were applied: ICAM-1 (GB11106, Servicebio, Wuhan, China, 1:1000 dilution), VCAM-1 (GB113498, Servicebio, 1:1000 dilution), and goat anti-rabbit HRP-labeled (GB23303, Servicebio, Wuhan, China, 1:200 dilution).

Mouse aortic tissue was sectioned (10 µm-thick, frozen) after 4% paraformaldehyde fixation. The frozen sections were blocked by 3% BSA for 30 min and then incubated in rabbit anti-CD68 antibody overnight. The slides were washed with PBS three times for five minutes each time. The tissues were coated with Cy3-goat anti-rabbit after a brief drying period. The obtained samples were then incubated in the dark for 40 min. After being stained with DAPI, the sections were sealed and stored away from light.

BMDMs were treated with 1 μg/mL Alexa Fluor 488 cholera toxin subunit B (CT-XB), and after washing with PBS, the cells were fixed under 4% paraformaldehyde for 15 min. After three washes with PBS, the cells were blocked with BSA (1% BSA, 22.52 mg/mL glycine, and 0.1% Triton X-100 in PBS) for 1 h and then treated with 1:500 anti-TLR9 primary antibody for 1 h. Following a one-hour incubation period with Alexa Fluor^®^ 647, the cells underwent three PBS washes. Confocal microscopy was used to capture representative images. The mean fluorescence intensity was calculated using Image J software.

### 2.10. Network Pharmacology Analysis

The SMILES identifier for artesunate was obtained from the PubChem database (https://pubchem.ncbi.nlm.nih.gov/), while the predicted target of ART was sourced from SwissTargetPrediction (http://swisstargetprediction.ch/). Targets for SLE and AS were extracted from GeneCards (https://www.genecards.org/) and OMIM (https://omim.org/databases). The overlapping targets were determined by intersecting the genes associated with SLE and AS. The shared targets between the disease and the putative target of ART were visualized using a Venn plot. Common targets were inputted into the STRING (https://cn.string-db.org/) database to construct a protein–protein interaction (PPI) network (species: *Homo sapiens*, medium confidence 0.4). Subsequently, the PPI data were imported into Cytoscape 3.9.1 for visualization. Gene Ontology (GO) enrichment analysis and Kyoto Encyclopedia of Genes and Genomes (KEGG) pathway enrichment analysis were performed using the DAVID database (https://david.ncifcrf.gov/home.jsp) to investigate the identified biological functions and potential mechanistic targets. The results were presented through bar graphs, bubble graphs, and chord graphs.

### 2.11. RT-qPCR Analysis

Using Trizol kits (Invivogen, San Diego, CA, USA), total RNAs were extracted from the aorta and kidney tissue according to the manufacturer’s instructions. Sangon Biotech (Shanghai, China) provided the primer sequences shown in Table 1. Evaluation of the CT methodology was conducted with GAPDH serving as the endogenous control.

### 2.12. Western Blot Analysis

Total protein was extracted from cells, isolated aorta, and kidney using RIPA lysis buffer (Biyuntian Inc., Hangzhou, China) supplemented with protease and phosphatase inhibitors. SDS-PAGE was employed to separate protein samples. Equal amounts of total protein (10 μg per lane, quantified by BCA assay) were separated by 7.5% SDS-PAGE. Next, the separated bands were subsequently mounted on membranes made of polyvinylidene fluoride (PVDF). After blocking the membrane with 5% skim milk for an hour, the membranes were treated with primary antibodies against TLR9 (#ab37154, Abcam Cambridge, MA, USA), MyD88 (#4283S, CST, Danvers, MA, USA), TRAF6 (#ab33915, Abcam Cambridge, MA, USA), p38 (#D13E1, CST, Danvers, MA, USA), pp38 (#D3F9, CST), ABCA1 (#ab66217, Abcam Cambridge, MA, USA)), ABCG1 (#ab52617, Abcam Cambridge, MA, USA)), PPARγ (#ab272718, Abcam Cambridge, MA, USA), SR-B1 (#ab217318, Abcam Cambridge, MA, USA, 1:2000 dilution), GAPDH (#ab8245, Abcam Cambridge, MA, USA, 1:1000 dilution), and β-actin (#4970S, CST, Danvers, MA, USA) overnight at 4 °C. The membranes were treated with appropriate secondary antibodies (#926-32211, LI-COR Biosciences, Lincoln, NE, USA) and (#926-68070, LI-COR Biosciences, Lincoln, NE, USA) for 2 h the following day. Protein expression levels were normalized to β-actin or GAPDH as loading controls. All experiments were performed in triplicate. Original western blots can be found at Supplementary Materials.

### 2.13. Statistical Analysis

The measurement results were obtained from at least three different experiments. Data were reported as mean ± SEM. The graphs were created using GraphPad Prism 8. A two-tailed Student’s *t*-test was performed to analyze the differences between the two groups. Difference among various groups was carried out using a one-way ANOVA, followed by Least Significant Difference (LSD), and a *p*-value < 0.05 was considered statistically significant.

## 3. Results

### 3.1. ART Attenuates Lupus-like Manifestations in Pristane-Treated ApoE^-/-^ Mice

**Figure 1:**
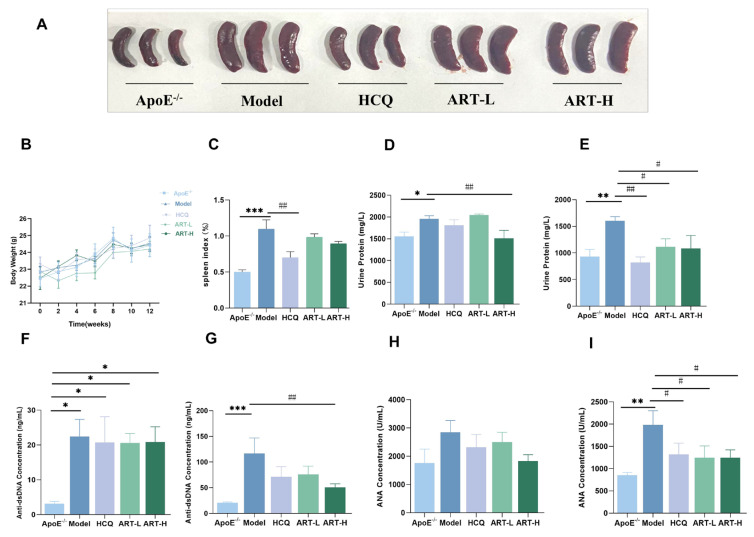
ART ameliorated the lupus-like phenotype in SLE-AS mice. (**A**) Gross appearance of mouse spleen. (**B**) Body weight was measured every two weeks after drug intervention. (**C**) Spleen index (Spleen weight/Body weight × 100%) in different experimental groups. (**D**,**E**) The expression levels of urine protein were determined at 4 and 12 weeks of gavage. (**F**) Analysis of the anti-dsDNA concentration four weeks after pristane injection and (**G**) 12 weeks post-gavage by ELISA. (**H**) ELISA for ANA concentration after four weeks of pristane injection. (**I**) ELISA for ANA concentration after 12 weeks of gavage. *** *p* < 0.001, ** *p* < 0.01, * *p* < 0.05 vs. ApoE^-/-^; ^##^ *p* < 0.01, ^#^ *p* < 0.05 vs. Model, *n* = 4–9 mice per group.

To model lupus-related atherosclerosis, pristane was intraperitoneally administered to atherosclerosis-prone ApoE^-/-^ mice. The body weight was measured at regular intervals following drug intervention, with no significant differences observed between groups (Figure 1B). The degree of splenomegaly was assessed using the splenic index (spleen weight/body weight 100%), revealing a significantly higher degree in the model group compared to the ApoE^-/-^ group. Treatment with HCQ notably mitigated this increase, while ART treatment tended to decrease splenomegaly (Figure 1A,C). Lupus nephritis, characterized by elevated urine protein and autoantibody levels, was evaluated for lupus diagnosis. Urine protein levels were measured at 4 and 12 weeks post-drug administration, showing a progressive increase in the model group compared to the ApoE^-/-^ group starting at four weeks (Figure 1D,E). HCQ and ART treatments reversed this trend (Figure 1D,E). Moreover, serum anti-dsDNA and ANA concentrations were determined to assess disease progression. Pristane-induced lupus-like manifestations in ApoE^-/-^ mice were associated with significant elevations in serum anti-dsDNA and ANA levels during disease progression. ART treatment effectively mitigated these effects (Figure 1F–I). Overall, our findings suggest that ART alleviates lupus-like manifestations in SLE-AS mice.

### 3.2. ART Attenuated Atherosclerotic Plaque Formation in SLE-AS Mice

**Figure 2:**
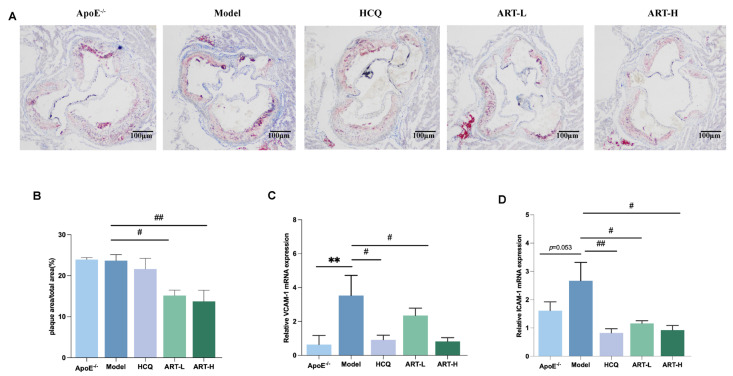
Artesunate reduced atherosclerotic lesions in SLE-AS mice. (**A**) Oil Red O staining, scale bar = 100 µm. (**B**) Quantitative analysis of the plaque area percentage of total area from ORO staining is shown in A. (**C**,**D**) qPCR analysis of VCAM-1 and ICAM-1 in the aortic tissue between groups. ** *p* < 0.01 vs. ApoE^-/-^; ^##^ *p* < 0.01, ^#^ *p* < 0.05 vs. Model, *n* = 3 mice per group.

To further understand the effect of ART on atherosclerotic plaque formation in SLE-AS mice, Oil Red O staining was used to analyze plaque area as a percentage of total aortic valve area and evaluate the severity of atherosclerosis. Our results indicate that ART can alleviate aortic plaque deposition to some extent in SLE-AS mice (Figure 2A). Vascular inflammatory markers, specifically VCAM-1 and ICAM-1, were quantified to elucidate ART’s anti-inflammatory properties. Both HCQ and ART treatment substantially decreased the mRNA expression of ICAM-1 and VCAM-1 in the aorta (Figure 2C,D). Overall, our findings suggest that ART reduces atherosclerotic plaque formation and suppresses the production of inflammatory factors.

### 3.3. ART Suppressed Kidney Inflammation in SLE-AS Mice

**Figure 3:**
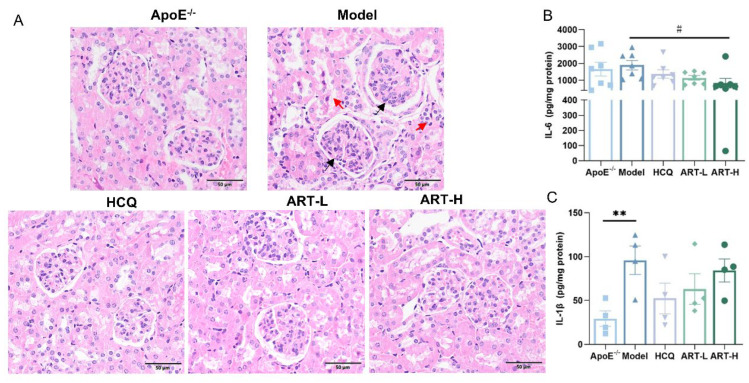
Artesunate alleviated kidney damage in SLE-AS mice. (**A**) Representative histochemical stain (HE stained) images of the kidney. Red arrows indicated inflammatory cell infiltration and edema degeneration of renal tubular epithelial cells. Black arrows indicated mesangial cell and endothelial cell proliferation. Original magnification, 20×. Scale bar, 50 μm. (**B**) ELISA for IL-1β and (**C**) IL-6 was performed using the kidney homogenate. ** *p* < 0.01 vs. ApoE^-/-^; ^#^ *p* < 0.05 vs. Model, *n* = 4–7 mice per group.

Although SLE and atherosclerosis originate through distinct pathways, their shared inflammatory effects on kidney tissue significantly contribute to progressive renal damage. Thus, we conducted HE staining of kidney tissues to examine the renal glomerular structure. Mice receiving ART treatment exhibited diminished glomerular growth, lesser growth of thylakoid and endothelial cells, decreased infiltration of inflammatory cells, and lesser edematous deterioration of renal tubular epithelial cells (Figure 3A). Next, we detected IL-6 and interleukin-1 beta (IL-1β) in kidney homogenates and found that the model group exhibited a significantly higher level of IL-1β than in the ApoE^-/-^ group (Figure 3B). After treatment with HCQ and ART, the concentration of IL-1β and IL-6 declined substantially (Figure 3B).

### 3.4. ART Inhibited Macrophage Foam Cell Accumulation and Induced Cholesterol Efflux in MDFC In Vivo and In Vitro

**Figure 4:**
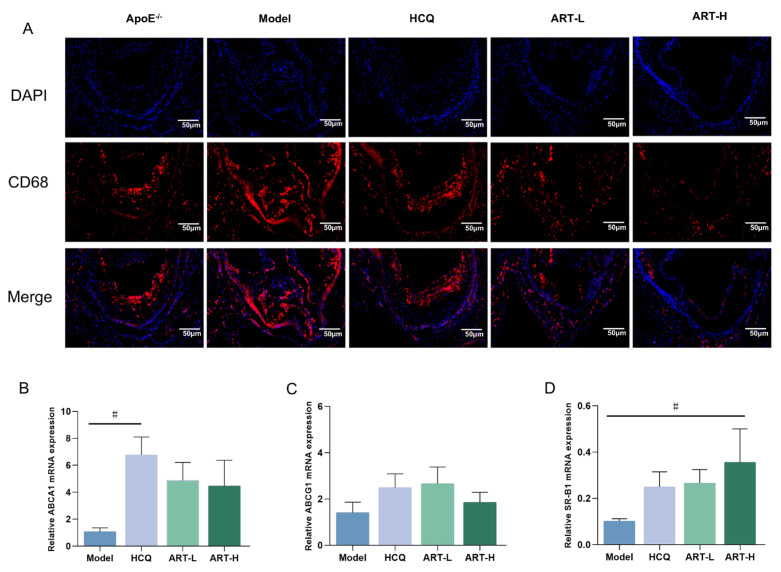
Artesunate ameliorated macrophage infiltration into the aorta. (**A**) Immunofluorescence images stained for CD68. Original magnification 2×. Scale bar, 50 μm. (**B**–**D**) Analysis of ABCA1, ABCG1, and SR-B1 mRNA expression in the aortic tissue. ^#^ *p* < 0.05 vs. Model, *n* = 3–4 mice per group.

Macrophage-mediated uptake of lipoproteins initiates foam cell formation, exacerbating inflammatory processes and promoting atherosclerotic plaque progression [23]. Immunofluorescence analysis of CD68-labeled mouse aortic macrophages revealed reduced macrophage infiltration following ART treatment (Figure 4A). Given the crucial roles of ABCA1, ABCG1, and SR-BI in cholesterol efflux and cellular cholesterol homeostasis, we assessed their expression in the aortic plaques of SLE-AS mice under ART treatment. Notably, ART treatment significantly upregulated the expression of ABCA1 and SR-BI genes compared to the model group (Figure 4B,D), while enhancing ABCG1 expression (Figure 4C).

**Figure 5:**
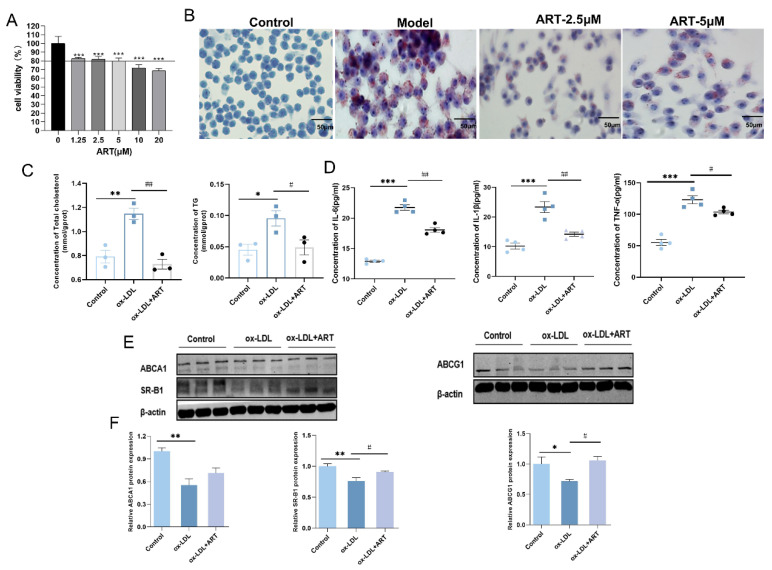
Artesunate reduced lipid accumulation by promoting cholesterol efflux in MDFC. (**A**) Analysis of cell viability across various concentrations of ART. (**B**) Representative images of lipid accumulation in ox-LDL-induced RAW264.7 cells treated with different concentrations of ART by Oil Red O. (**C**) Total cholesterol (TC) and triglyceride (TG) content in MDFC. (**D**) Analysis of culture supernatants to quantify IL-6, IL-1β, and TNF-α secretion by ELISA. (**E**,**F**) Relative protein levels of ABCA1, ABCG1, and SR-B1, with β-actin as a loading control (original images can be found in Figure S2). *** *p* < 0.001, ** *p* < 0.01, * *p* < 0.05 vs. Control; ^##^ *p* < 0.01, ^#^ *p* < 0.05 vs. ox-LDL.

To further elucidate the effects of ART on MDFC, an in vitro foam cell model was established by inducing RAW264.7 cells with ox-LDL. Initially, a CCK-8 assay was conducted to assess the impact of ART on cell viability. Results indicated that ART concentrations below 10 μM yielded fewer adverse effects on cell viability (Figure 5A). Subsequently, RAW264.7 cells exposed to ox-LDL for 24 h exhibited intense red staining, indicative of lipid accumulation (Figure 5B). Notably, ART at a concentration of 2.5 μM significantly reduced lipid deposition compared to 5 μM, prompting the selection of 2.5 μM for subsequent experiments. Further analysis revealed that ART treatment markedly decreased intracellular levels of total cholesterol (TC) and triglycerides (TG) in MDFC (Figure 5C). Moreover, pretreatment with ART substantially attenuated the production of pro-inflammatory cytokines, including IL-6, TNF-α, and IL-1β, in ox-LDL-exposed RAW264.7 cells (Figure 5D). Western blot analysis demonstrated that ox-LDL significantly downregulated the expression of ABCA1, ABCG, and SR-B1 in MDFC. However, ART treatment effectively counteracted these changes, restoring the expression levels of these cholesterol efflux regulators (Figure 5E,F). In summary, these findings suggest that ART modulates cholesterol efflux by upregulating the expression of ABCA1, ABCG1, and SR-B1, thereby mitigating lipid accumulation and inflammation in MDFC.

### 3.5. ART Inhibited TLR9 Recruitment to Lipid Rafts and Suppressed the Inflammation Pathway

**Figure 6:**
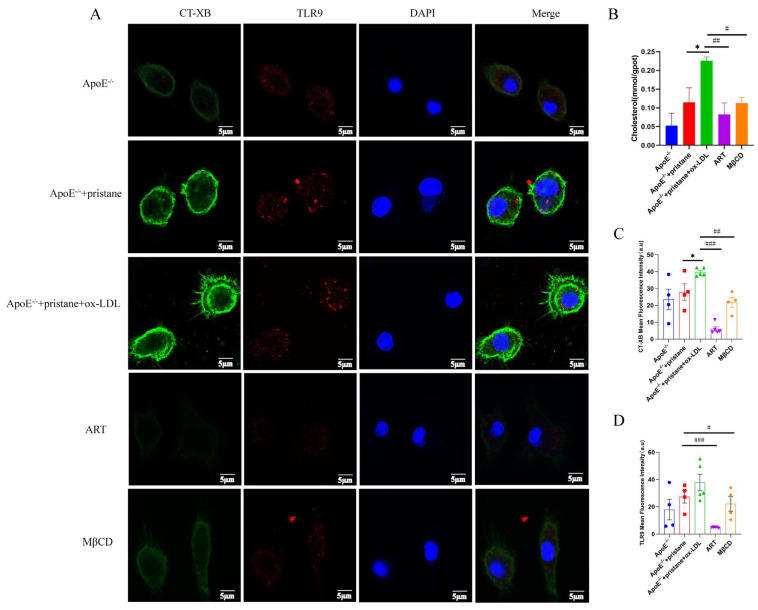
ART inhibits the ox-LDL-induced recruitment of TLR9 into lipid rafts. BMDMs were pretreated with ART (2 μM, 6 h) and MβCD (2 μM, 40 min), then stimulated with ox-LDL (40 μg/mL, 24 h). (**A**) Total cholesterol (TC) in bone marrow macrophages. (**B**) Confocal microscopy was used to investigate the cells after immunostaining with FITC-conjugated CT-XB for lipid rafts (green) and anti-TLR9 primary antibody and Alexa Fluor 647-conjugated secondary antibody for TLR9 (red). (**C**) Quantification of CT-XB and (**D**) TLR9 mean fluorescence intensity from 4–5 independent experiments. * *p* < 0.05 vs. ApoE^-/-^+pristane; ^#^ *p* < 0.05, ^##^ *p* < 0.01, ^###^ *p* < 0.001 vs. ApoE^-/-^+pristane+ox-LDL.

Prior studies have demonstrated that the absence of ABCA1 leads to the accumulation of cholesterol in lipid rafts, facilitating the recruitment of TLR9 and its cognate adaptor proteins, MyD88, into lipid rafts. This process results in the secretion of inflammatory factors [24,25]. To further investigate this mechanism, BMDMs were treated with or without ox-LDL in the presence or absence of ART or MβCD, a cholesterol-depleting agent. Elevated cholesterol levels were observed in ox-LDL-induced BMDMs, and ART treatment significantly reduced cholesterol accumulation (Figure 6B). We performed immunofluorescence staining using the lipid raft marker CT-XB to assess lipid raft aggregation (Figure 6A). The results revealed that ox-LDL stimulation markedly increased the intensity of green fluorescence in labeled lipid rafts, indicating enhanced lipid raft formation in BMDMs. However, pretreatment with ART or MβCD effectively inhibited this intense fluorescence, suggesting a reduction in lipid raft aggregation (Figure 6C). Moreover, ox-LDL promoted TLR9 recruitment to lipid raft domains; however, pretreatment with ART or MβCD effectively inhibited this intense fluorescence, suggesting a reduction in TLR9 recruitment (Figure 6D). Collectively, these findings demonstrate that ART reduces cholesterol enrichment in lipid rafts and inhibits the recruitment of TLR9 into these domains, thereby potentially attenuating the inflammatory response associated with lipid raft-mediated signaling pathways.

**Figure 7:**
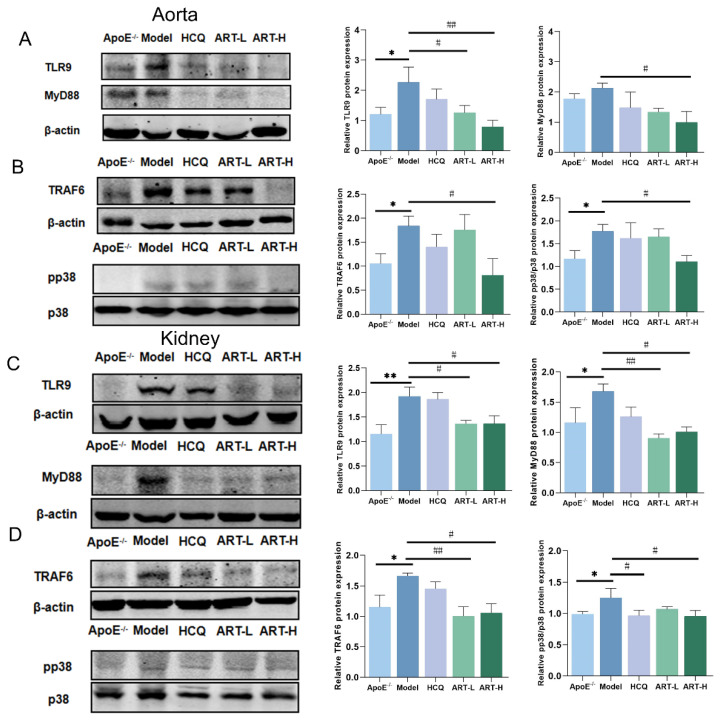
Artesunate decreased TLR9, MyD88, TRAF6, and pp38 expression in the kidney and aorta tissue of SLE-AS mice. (**A**,**B**) Western blot analysis of the expression of TLR9, MyD88, TRAF6, and pp38 in aorta tissue (original images can be found in Figure S3). (**C**,**D**) Western blot analysis of the expression of TLR9, MyD88, TRAF6, and pp38 in kidney tissue (original images can be found in Figure S4). ** *p* < 0.01, * *p* < 0.05 vs. ApoE^-/-^; ^##^ *p* < 0.01, ^#^ *p* < 0.05 vs. Model, *n* = 3–4 mice per group.

**Figure 8:**
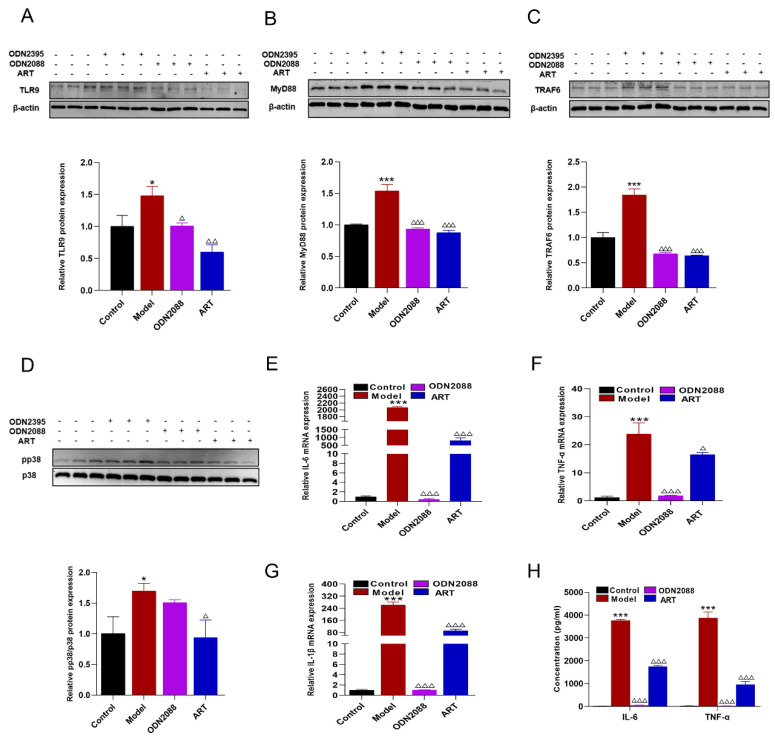
Inflammatory inhibitory effect of ART on RAW 264.7 macrophages. (**A**–**D**) Protein levels of TLR9, MyD88, TRAF6, and phospho-p38 (pp38) were assessed by immunoblotting in RAW 264.7 macrophages pretreated with ART or ODN2088 and subsequently stimulated with ODN2395. β-actin served as an internal control (original images can be found in Figure S5). (**E**–**G**) mRNA levels of IL-6, TNF-α, and IL-1β were analyzed using qPCR followed by ART or ODN2088. (**H**) Macrophages were pretreated with ART or ODN2088 and stimulated with ODN2395, IL-6, and TNF-α were performed using cellular supernatant. Concentration: ODN2395 (2 μM), ODN2088 (1 μM), ART (2.5 μM). The bar graphs represent the analysis from three independent experiments. *** *p* < 0.001, * *p* < 0.05 vs. Control; ^△△△^ *p*< 0.001, ^△△^ *p*< 0.01, ^△^ *p* < 0.05 vs. Model.

TLR9 signaling is known to be initiated upon the rapid trafficking of TLR9 to lipid rafts in response to specific ligands, subsequently leading to the production of pro-inflammatory factors [26,27,28]. To further elucidate the role of ART in modulating this pathway, Western blot analysis was performed. The results demonstrated that the expression levels of TLR9, MyD88, TRAF6, and phosphorylated p38 (pp38) were significantly upregulated in the aorta of SLE-AS mice compared to the ApoE^-/-^ group. However, ART administration restored these protein levels, indicating a regulatory effect of ART on the TLR9 signaling pathway (Figure 7A,B). A similar restorative effect of ART was observed in renal tissues (Figure 7C,D). RAW 264.7 cells were stimulated with ODN2395 to establish an in vivo inflammatory model. ART significantly downregulated TLR9, MyD88, TRAF6, and p38 protein levels in ODN2395-treated RAW 264.7 cells (Figure 8A–D). At the same time, ODN2395 stimulation markedly upregulated the secretion of pro-inflammatory cytokines, including IL-6, TNF-α, and IL-1β, compared to the control groups. These inflammatory responses were significantly attenuated by both the TLR9 inhibitory oligonucleotide (ODN2088) and ART treatment (Figure 8E–H). Overall, these results revealed that ART mitigates inflammation by suppressing TLR9 signaling pathways.

### 3.6. Network Pharmacology Analysis on ART Treatment of SLE-AS Disease

**Figure 9:**
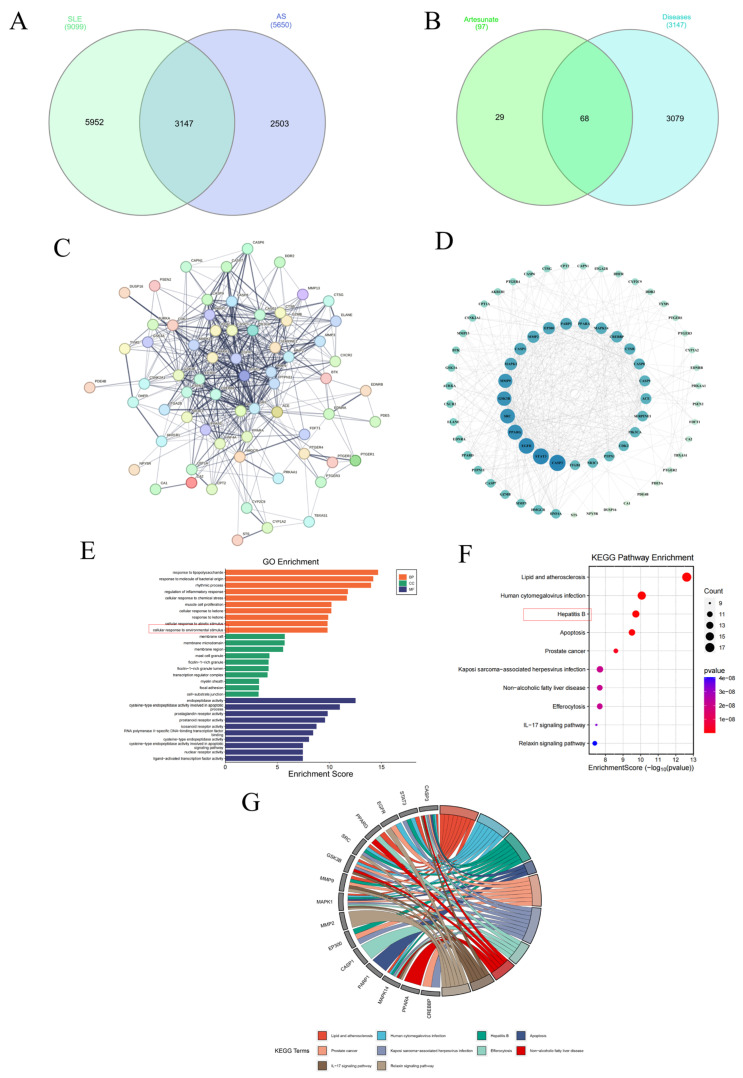
Network pharmacology analysis of ART therapeutic targets for SLE-AS. (**A**) Venn plot showing the intersections of SLE- and AS-related genes. (**B**) Venn plot of SLE-AS-related targets and ART targets. (**C**,**D**) PPI network of the common targets of ART and AS-related genes. (**E**) GO analysis of SLE-AS and ART targets. (**F**,**G**) Bubble chart depicting the KEGG pathway enrichment analysis of ART-SLE-AS genes and chord plot of the core targets involved in the top ten pathways.

To further explore the molecular mechanisms of ART regulating cholesterol homeostasis, inhibiting inflammation, and preventing the progression of SLE-AS. Network pharmacology was conducted. In the GeneCards database, 9040 SLE-related genes and 5649 AS-related genes were found. In this study, 99 SLE- and 3 AS-related genes were derived from the OMIM database. After duplication removal, 9099 CD-related and 5650 AS-related genes were finally selected (Figure 9A). The SLE and AS disease targets were intersected, and 3147 common target genes were screened (Figure 9A). The intersection of disease-related genes and drug-active ingredient-related targets was obtained, yielding 68 genes (Figure 9B). Subsequently, we imported intersecting targets into the String11.0 platform to obtain interactions between the targets (Figure 9C). The PPI network-based analysis was conducted to identify common drug–disease targets. The top 10 targets based on degree value included *CASP3*, *STAT3*, *EGFR*, *PPARG*, *SRC*, *GSK3B*, *MMP9*, *MAPK1*, *MMP2*, and *EP300* (Figure 9D). GO and KEGG enrichment analyses were performed on ART targets in treating SLE complicated by AS in mice models. GO analysis showed predominant involvement in the inflammatory response, while KEGG pathway enrichment analysis indicated lipid and atherosclerosis as the significantly affected pathways (Figure 9E,F). A KEGG chord plot illustrated the involvement of *CASP3*, *STAT3*, *EGFR*, *PPARG*, *SRC*, *GSK3B*, *MMP9*, *MAPK1*, *MMP2*, *EP300*, *CASP1*, *PARP1*, *MAPK14*, *PPARA*, and *CREBBP* in the top ten KEGG terms (Figure 9G).

### 3.7. ART Inhibited Macrophage Foam Cell Formation by Upregulating the PPARγ-ABCA1/ABCG1/SR-B1 Pathway

**Figure 10:**
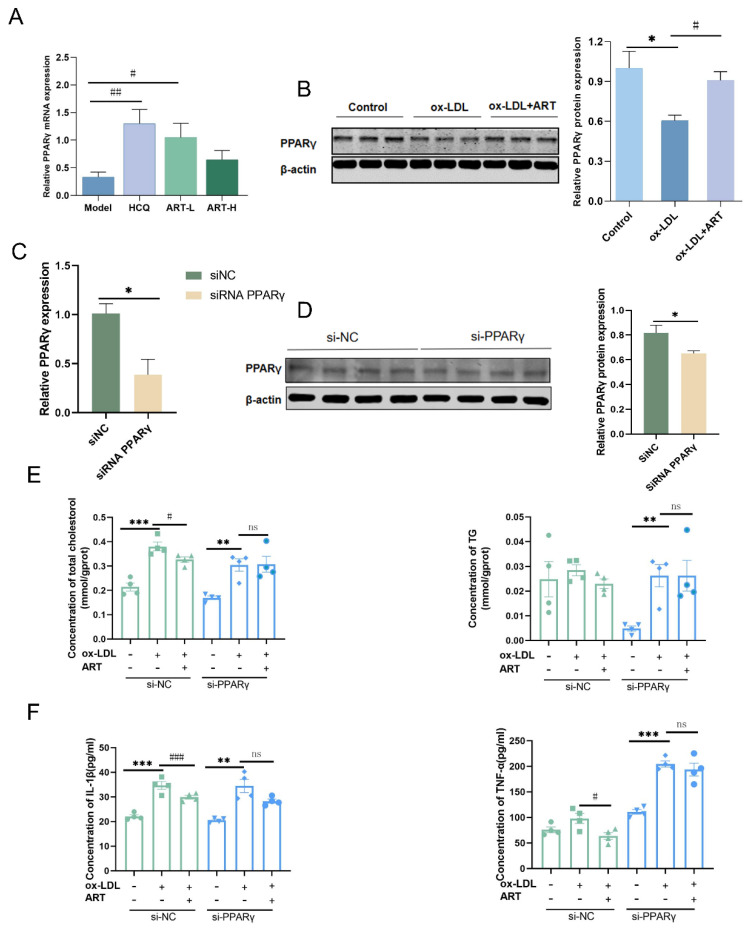
ART upregulated PPARγ expression. (**A**) The relative expression level of PPARγ in SLE-AS mice. (**B**) Western blot analysis of PPARγ in MDFC treated with ART (original images can be found in Figure S6). Relative mRNA (**C**) and protein (**D**) of PPARγ in macrophages transfected with si-NC and si-PPARγ (original images can be found in Figure S7). (**E**) After si-NC or si-PPARγ transfection, intracellular TG and TC levels. (**F**) After si-NC or si-PPARγ transfection, IL-1β and TNF-α were assessed by ELISA. ^###^ *p* < 0.001, ^##^ *p* < 0.01, ^#^ *p* < 0.05 vs. Model. *** *p* < 0.001, ** *p* < 0.01, * *p* < 0.05. ns, not significant.

**Figure 11:**
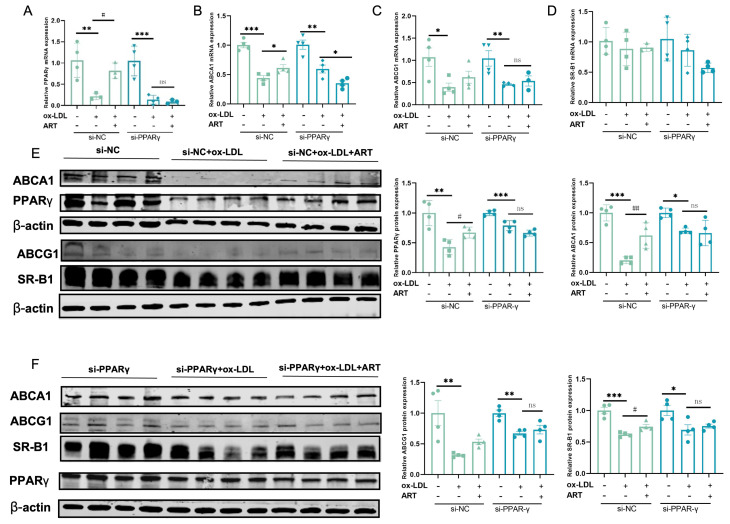
Ablation of PPARγ attenuated the ART-induced cholesterol efflux in MDFC. (**A**–**D**) After si-NC or si-PPARγ transfection, the relative mRNA of PPARγ, ABCA1, ABCG1, and SR-B1 was detected in the presence or absence of ART. (**E**,**F**) After si-NC or si-PPARγ transfection, protein expression levels of PPARγ, ABCA1, ABCG1, and SR-B1 were detected in the presence or absence of ART. β-actin served as an internal control (original images can be found in Figure S8). *** *p* < 0.001, ** *p* < 0.01, * *p* < 0.05; ^##^ *p* < 0.01, ^#^ *p* < 0.05; ns, not significant.

Notably, PPARG (PPARγ) was identified within the top 10 KEGG pathway intersections of ART and SLE-AS targets, and PPARγ plays a crucial role in regulating lipid metabolism and maintaining cholesterol homeostasis [29,30]. PPARγ, as a central regulator, orchestrates cholesterol efflux through the LXRα-ABCA1/ABCG1 signaling axis, prompting its selection as a primary target for further experimental validation. Downregulation of PPARγ was observed in both MDFC and aortic tissues of SLE-AS mice (Figure 10A,B). In contrast, ART treatment significantly enhanced PPARγ expression in both in vivo and in vitro models. Next, PPARγ’s impact on ART-induced cholesterol efflux and MDFC formation was investigated. We examined the function of ART on MDFC using small interfering RNAs (siRNAs) targeting PPARγ (si-PPARγ) and a negative control siRNA (si-NC), with the knockdown efficiency assessed through qRT-PCR and Western blotting. As shown in Figure 10C,D, si-PPARγ significantly decreased PPARγ mRNA and protein expression in MDFC, unlike si-NC. Subsequent experiments revealed that PPARγ knockdown abolished the ART-mediated reduction in TC and TG, as well as the decreased secretion of pro-inflammatory cytokines IL-1β and TNF-α in MDFC (Figure 10E,F). Moreover, the knockdown of PPARγ significantly inhibited the increased mRNA and protein expression of PPARγ, ABCA1, ABCG1, and SR-B1 in MDFC treated with ART (Figure 11A–E). In summary, these results collectively indicate that ART inhibits MDFC formation by augmenting cholesterol efflux by activating the PPARγ-ABCA1/G1/SR-B1 pathway.

## 4. Discussion

SLE is a chronic autoimmune disease often associated with an increased risk of AS. The dysregulation of cholesterol metabolism and the pro-inflammatory environment inherent to SLE contribute to the accelerated progression of atherosclerotic disease [31,32]. Consequently, identifying effective therapeutic agents for the prevention and treatment of SLE-AS is of paramount importance. Artesunate, a derivative of artemisinin, has a well-established pharmacological profile and has shown promising efficacy in treating SLE and other inflammatory diseases. Building on the demonstrated effectiveness of Qinghao decoction in managing SLE-AS, our study sought to elucidate the underlying therapeutic mechanisms by conducting comprehensive in vivo and in vitro experiments focusing on its active ingredient, artemisinin.

Establishing a reliable SLE-AS mouse model is crucial for studying its pathogenesis. Currently, while several mouse models of SLE-AS exist, they often suffer from limitations such as limited efficacy, prolonged breeding, and complex purification processes [33,34,35]. Among existing approaches, the induction of lupus via pristane injection in ApoE^-/-^ mice remains a widely utilized method for generating SLE-AS models [21,36,37]. Pristane, an isoprene alkane, enhances the autoimmune response, triggers the production of various autoantibodies, and induces lupus-like symptoms [38]. Apolipoprotein E is a vital element of chylomicron, VLDL, and HDL, and it regulates lipid metabolism balance [39,40]. Deletion of the *ApoE* gene results in high plasma cholesterol levels and cholesterol accumulation in the walls of blood vessels, ultimately resulting in the development of AS [41]. Feeding ApoE^-/-^ mice a high-fat diet (HFD) can accelerate the progression of AS, making ApoE^-/-^ mice a widely used model for studying spontaneous atherosclerosis [42]. In the present study, ApoE^-/-^ mice were intraperitoneally injected with pristane and fed an HFD for 16 weeks to establish a disease model. Following pristane injection, we observed lupus-like symptoms, including splenomegaly, elevated urinary protein levels, increased serum autoantibodies (anti-dsDNA and ANA), and renal injury characterized by glomerular hypertrophy, proliferation of mesangial and endothelial cells, and edema degeneration of renal tubular epithelial cells. Moreover, Oil Red O staining revealed that discernible plaque deposition was observed in the model group. Our results illustrated the successful establishment of a robust model of SLE-AS in ApoE^-/-^ mice by pristane injection.

In SLE, dysregulated immune responses can result in inflammation and vascular damage. It has been reported that IL-1β is the main factor that increases adhesion molecules and IL-6 levels in endothelial cells [43]. Consistent with previous studies, our research discovered elevated pro-inflammatory cytokine levels in the kidneys of SLE-AS mice. Moreover, the engulfment of ox-LDL by macrophages results in lipid accumulation and the development of foam cells, which is a characteristic of atherosclerosis [44,45,46]. Previous research has revealed that ART alleviates obesity caused by abnormal lipid metabolism through its anti-inflammatory and hypolipidemic effects [47]. In the current study, ART treatment was found to attenuate atherosclerotic plaque formation in the aortic intima of pristane-induced ApoE^-/-^ mice. While the immunohistochemical staining patterns suggest a trend toward reduced VCAM-1/ICAM-1 expression in ART-treated groups, the pale staining intensities (Figure S1) preclude definitive conclusions. This limitation is not uncommon in vascular immunohistochemistry studies due to inherent tissue heterogeneity [48]. Importantly, the concomitant mRNA expression data provide evidence for the anti-inflammatory effects. We provided evidence that ART downregulated ICAM-1 and VCAM-1 levels in SLE-AS mice and reduced macrophage infiltration in aortic plaques.

The maintenance of cholesterol homeostasis relies on the balance of different cellular responses to cholesterol. Since most cells in the body do not have sufficient capacity to degrade cholesterol, this imbalance, especially the obstruction of excretion, will cause free cholesterol to accumulate in cells, foam cells to foam, and eventually lead to atherosclerosis. Therefore, reducing intracellular cholesterol accumulation by promoting cholesterol efflux is an effective mechanism to prevent AS. In macrophages, the proteins ABCA1, ABCG1, and SR-B1 mediate cholesterol efflux transport. Recent studies have shown that atherosclerosis is accelerated and foam cell production is increased when macrophage ABCA1/G1 is deficient [49,50]. In addition, the absence of macrophage ABCA1/G1 promotes atherosclerosis by increasing pro-inflammatory cytokine production in atherosclerotic plaques [51]. Our experimental data demonstrate that 24-h ox-LDL stimulation downregulates ABCA1 expression in macrophages, which appears inconsistent with some previous studies reporting ox-LDL-induced activation of the LXR/ABCA1 pathway. However, this discrepancy likely stems from differences in experimental conditions, particularly exposure duration and ox-LDL concentration. While most studies showing ABCA1 upregulation employed short-term (6–12 h), our long-term (24 h) stimulation model better mimics the persistent ox-LDL exposure characteristic of advanced atherosclerotic lesions. Notably, prolonged ox-LDL treatment in our study triggered significant release of pro-inflammatory cytokines, including TNF-α, IL-6, and IL-1β (Figure 5D), establishing a critical link between sustained ox-LDL exposure, inflammation, and ABCA1 suppression. These findings highlight a pathological shift from protective cholesterol efflux to pro-inflammatory responses in advanced atherosclerosis [52], suggesting stage-specific therapeutic strategies are needed to effectively target ABCA1 dysfunction. This study found that ART could markedly increase the expression of ABCA1, ABCG1, and SR-B1 in SLE-AS mice. In vitro experiments demonstrated that ART intervention inhibited MDFC formation, reduced cholesterol accumulation, and suppressed inflammatory factors. These findings collectively suggested that ART exerts a beneficial effect on AS treatment.

Lipid rafts are sphingolipid- and cholesterol-rich specialized microdomains on the plasma membrane that are essential for signal transduction [53,54]. It has been shown that the recruitment of TLR9 to lipid rafts depends on cholesterol involvement. TLR9 recruitment to lipid rafts is critical for TLR9 activation during the initial stages of inflammation [55,56]. Inflammation plays a central role in the pathogenesis of AS [57]. In the development of atherosclerosis, TLR9 activation in immune cells, particularly macrophages, triggers the production of pro-inflammatory cytokines. Importantly, our results showed that ART could enhance cholesterol efflux and suppress the lipid raft-mediated TLR9 inflammatory signaling pathway.

Network pharmacological analyses revealed a significant relationship between PPARγ and ART in the treatment of SLE-AS. Activation of PPARγ is known to be critical in maintaining cholesterol homeostasis and inhibiting foam cells and atherosclerotic plaque formation. PPAR-γ agonists are widely used in the treatment of metabolic and inflammatory diseases, including type 2 diabetes, atherosclerosis, and non-alcoholic fatty liver disease (NAFLD) [58,59]. While these drugs demonstrate significant therapeutic benefits, their clinical application is limited by several drawbacks and potential adverse effects [60]. ART improved lipid metabolism and suppressed inflammation in SLE-AS mice. Furthermore, it has been shown that PPARγ augments ABCA1/G1 and SR-B1 expression to inhibit MDFC formation and exert an anti-atherosclerosis effect. This study consistently showed that ART boosted PPARγ levels and upregulated ABCA1, ABCG1, and SR-B1 expression. Remarkably, inhibiting PPARγ with siRNA effectively reversed ART’s impact on PPARγ, hindering the enhancement of ABCA1, ABCG1, and SR-B1, along with cholesterol buildup and the secretion of inflammatory molecules. Our results showed that by upregulating the PPARγ-ABCA1/ABCG1/SR-B1 pathway, ART could enhance cholesterol efflux and ameliorate inflammation, thereby alleviating atherosclerosis.

In conclusion, our findings provide preliminary evidence of the therapeutic potential of ART in SLE-AS mice. Mechanistically, ART can promote cholesterol efflux through the upregulation of the PPARγ-ABCA1/ABCG1/SR-B1 pathway while inhibiting the lipid raft-organized TLR9/MyD88 inflammatory pathway.

## 5. Conclusions

This study demonstrates that ART alleviates SLE-AS by restoring cholesterol homeostasis via PPARγ-mediated upregulation of ABCA1/ABCG1/SR-B1, enhancing macrophage cholesterol efflux and reducing foam cell formation. Additionally, ART suppresses TLR9 recruitment to lipid rafts, inhibiting downstream inflammatory signaling. These dual mechanisms—modulating lipid metabolism and dampening TLR9-driven inflammation—explain ART’s protective effects against lupus-associated atherosclerosis. Given the lack of targeted therapies for SLE-AS, ART’s ability to simultaneously address dyslipidemia and autoimmunity highlights its therapeutic potential. Further studies should validate these findings in clinical settings and explore ART’s synergy with existing SLE treatments. Our results provide new insights into SLE-AS pathogenesis and suggest ART as a promising multi-target intervention.

## Figures and Tables

**Table 1:** Primer sequences used in this study.

Gene	Forward Sequence	Reverse Sequence
GAPDH	5′-AGGTCGGTGTGAACGGATTTG-3′	5′-TGTAGACCATGTAGTTGAGGTCA-3′
IL-6	5′-GGCCTTCCCTACTTCACAAG-3′	5′-ATTTCCACGATTTCCCAGAG-3′
TNF-α	5′-GACTAGCCAGGAGGGAGAACAGA-3′	5′-CCTGGTTGGCTGCTTGCTT-3′
IL-1β	5′-CAACCAACAAGTGATATTCTCCATG-3′	5′-GATCCACACTCTCCAGCTGCA-3′
ABCA1	5′-GCATTGTCAAGGAGGGGAGAT-3′	5′-CTTCAGGTCAGGGTTGGAGC-3′
ABCG1	5′-GTCTGAACTGCCCTACCTACCA-3′	5′-AAAGAAACGCCTTCACATCG-3′
PPAR-γ	5′-CCCACAGAGAAGGAAGACCA-3′	5′-ACCACAGCACAGGACATTCA-3′
SR-B1	5′-GCAAATTTGGCCTGTTTGTT-3′	5′-GATCTTGCTGAGTCCGTTCC-3′
ICAM-1	5′-TTCACACTGAATGCCAGCTC-3′	5′-GTCTGCTGAGACCCCTCTTG-3′
VCAM-1	5′-GCCCATCCTCTGTGACTCAT-3′	5’-AGGCCACAGGTATTTTGTCG-3’

## Data Availability

The original contributions presented in this study are included in this article/Supplementary Materials. Further inquiries can be directed to the corresponding author(s).

## Supplementary Materials

The following supporting information can be downloaded at: https://www.mdpi.com/article/10.3390/biom15081078/s1. Figure S1: The effect of ART intervention on the expression of VCAM-1 and ICAM-1 in SLE-AS mice. Representative immunostaining for VCAM-1 and ICAM-1 in the aortic sinus sections.

## Abbreviations

The following abbreviations are used in this manuscript:

ABCA1/ABCG1	ATP-binding cassette transporters A1/G1
ApoE^-/-^	apolipoprotein E knockout
ART	artesunate
AS	atherosclerosis
CVD	cardiovascular disease
CCK-8	cell counting kit
FC	free cholesterol
CT-XB	cholera toxin subunit
ICAM-1	intercellular cell adhesion molecule-1
MDFC	macrophage-derived foam cells
MyD88	myeloid differentiation primary-response protein 88
ox-LDL	oxidized low-density lipoprotein
PPARγ	proliferator-activated receptor gamma
SLE	systemic lupus erythematosus
SR-B1	scavenger receptor-B1
TLR9	Toll-like receptor
TRAF6	tumor necrosis factor receptor-associated factor 6
VCAM-1	vascular cell adhesion molecule-1
