# Supplementary material for: Artesunate Ameliorates SLE Atherosclerosis Through PPARγ-Driven Cholesterol Efflux Restoration and Disruption of Lipid Raft-Organized TLR9/MyD88 Signaling Pathway
Source: Biomolecules. 2025 Jul 25;15(8):1078. doi: 10.3390/biom15081078 (PMC12383290; doi:10.3390/biom15081078)
Supplement: Supplementary file 1 [file biomolecules-15-01078-s001.zip › biomolecules-3713969-supplementary.pdf]

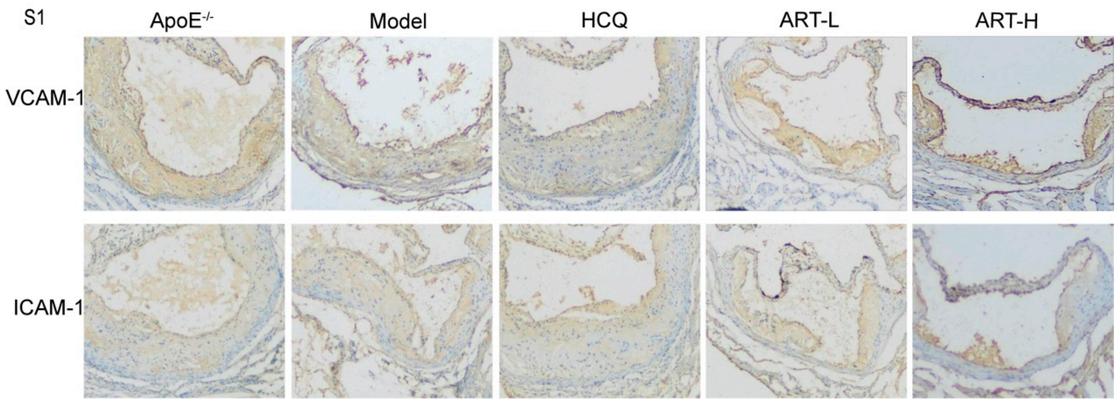

Figure S1 The effect of ART intervention on the expression of VCAM-1 and ICAM-1 in SLE-AS mice Representative immunostaining for VCAM-1 and ICAM-1 in the aortic sinus sections.
