# Supplementary material for: Artesunate Ameliorates SLE Atherosclerosis Through PPARγ-Driven Cholesterol Efflux Restoration and Disruption of Lipid Raft-Organized TLR9/MyD88 Signaling Pathway
Source: Biomolecules. 2025 Jul 25;15(8):1078. doi: 10.3390/biom15081078 (PMC12383290; doi:10.3390/biom15081078)
Supplement: Supplementary file 1 [file biomolecules-15-01078-s001.zip › Western blot.pdf]

ox-LDL induced macrophage n=3

Figure S2

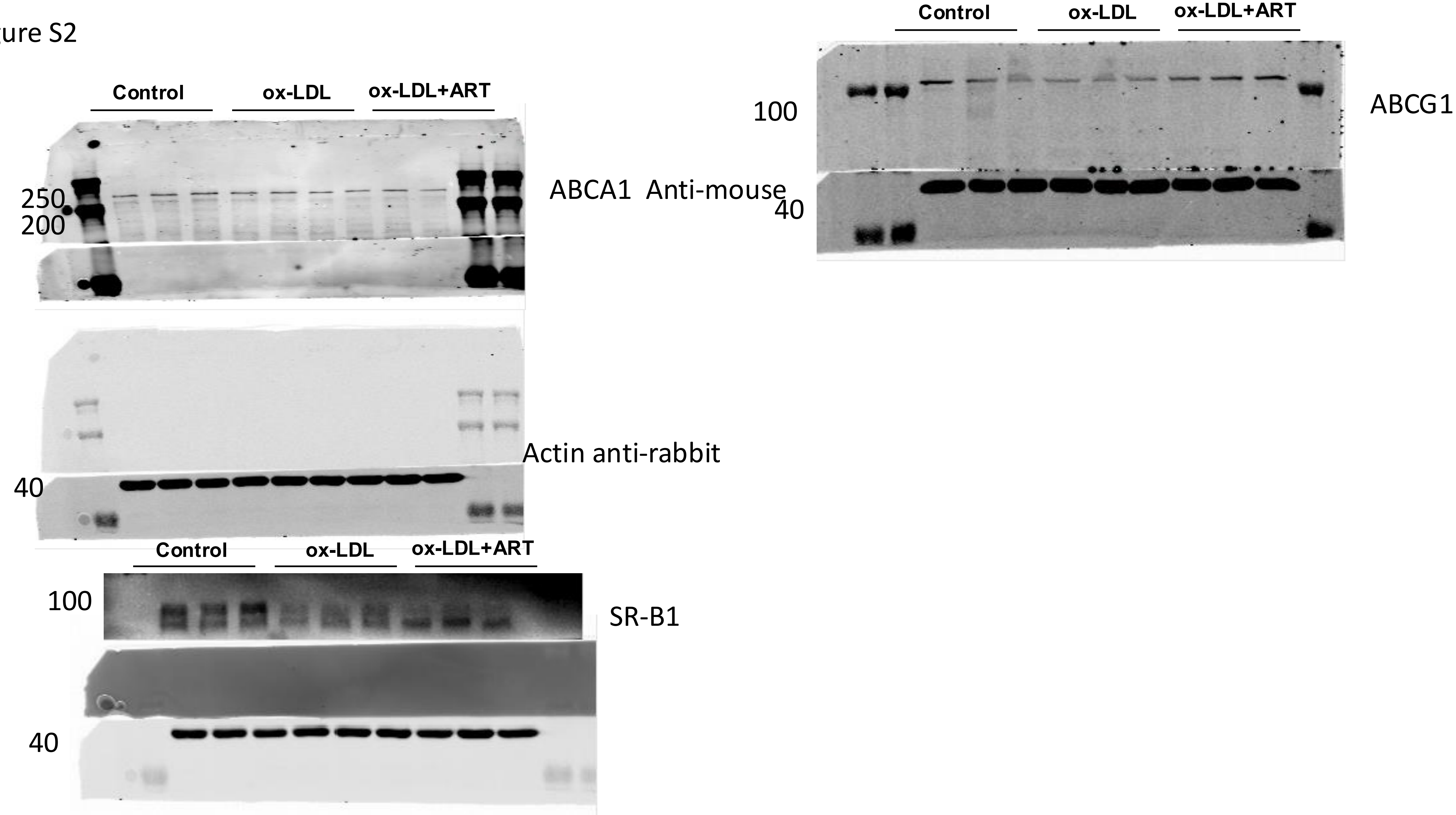

Figure S3

Aortic

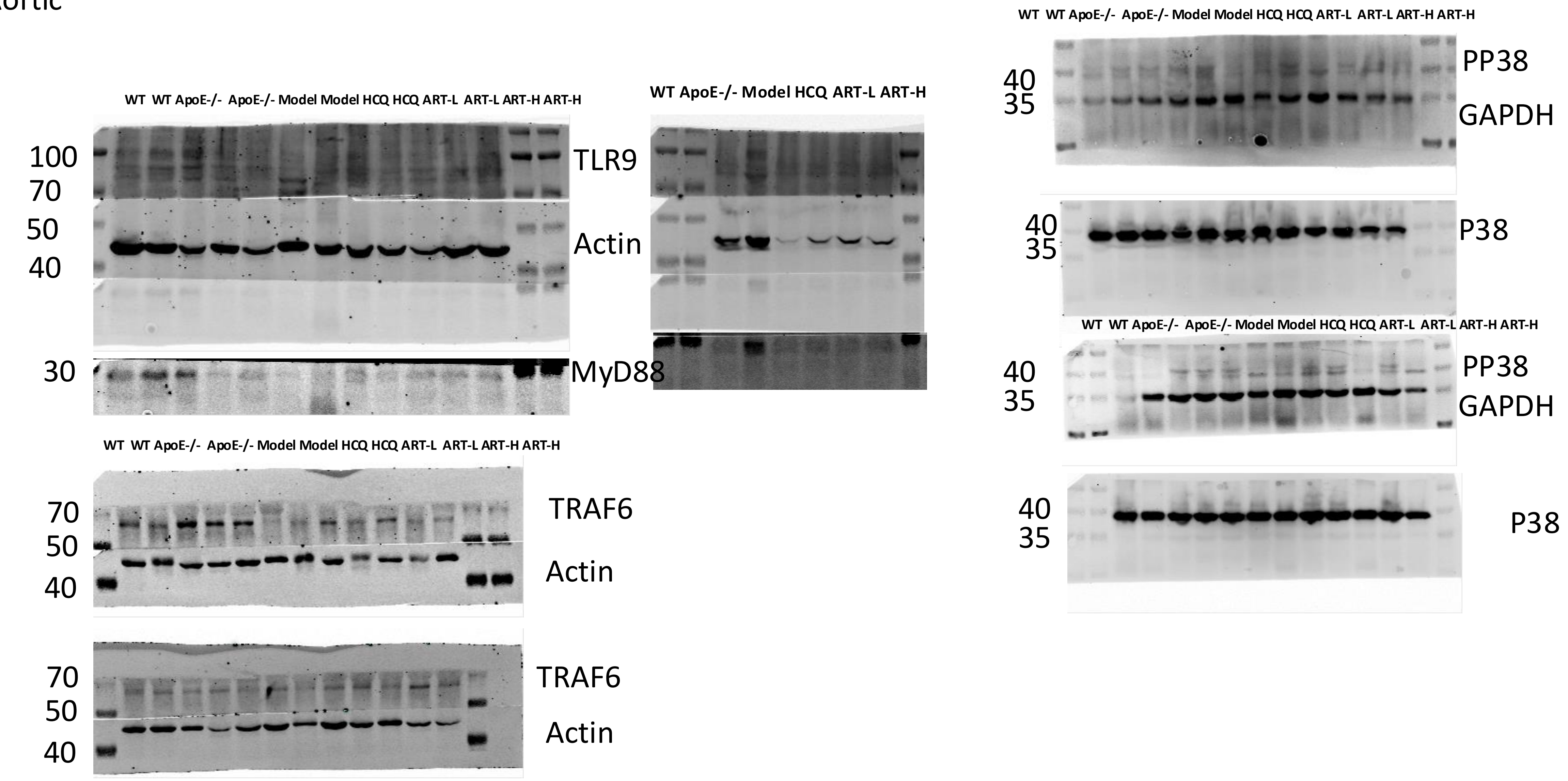

Figure S4
kidney

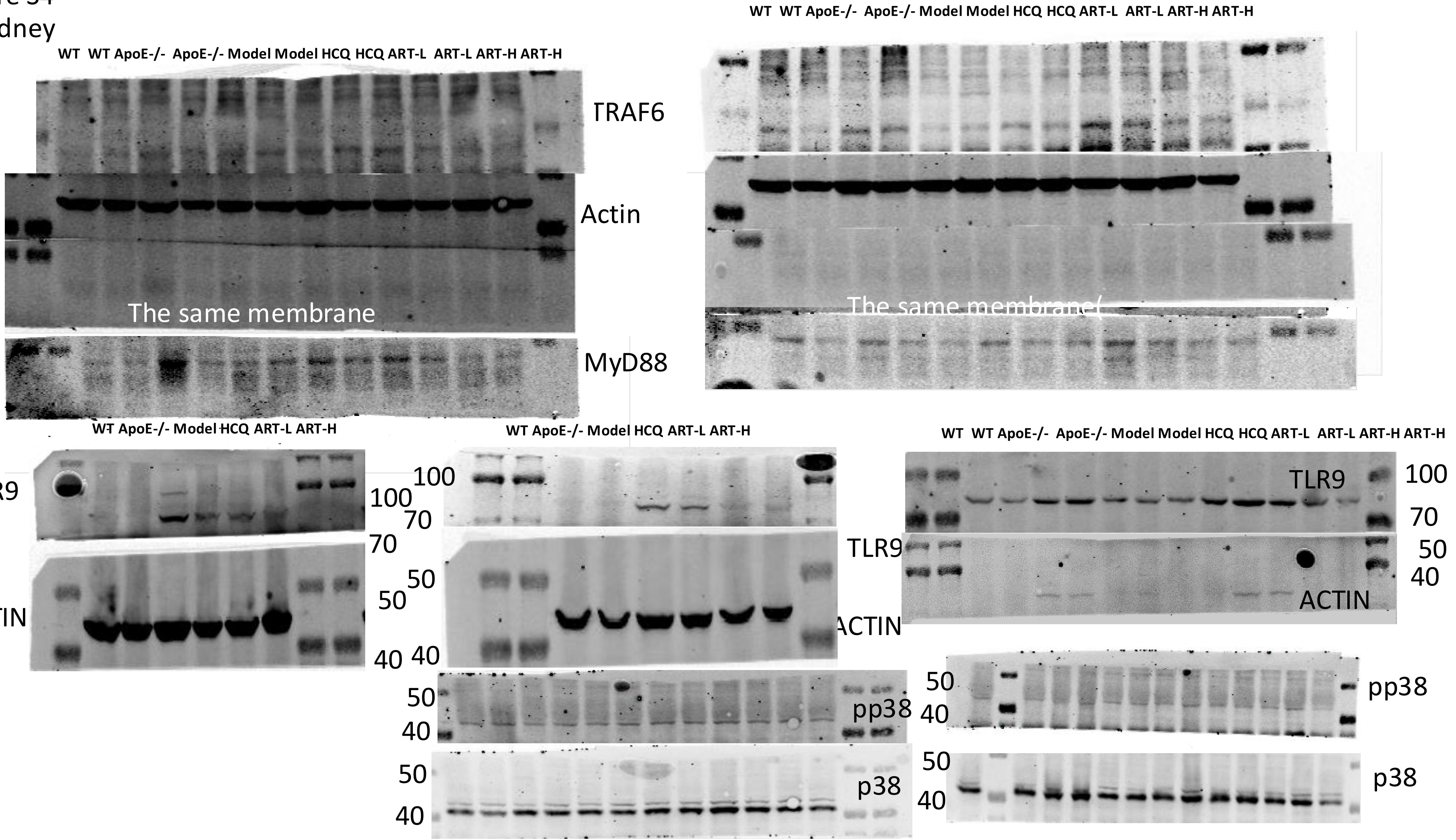

Figure S5

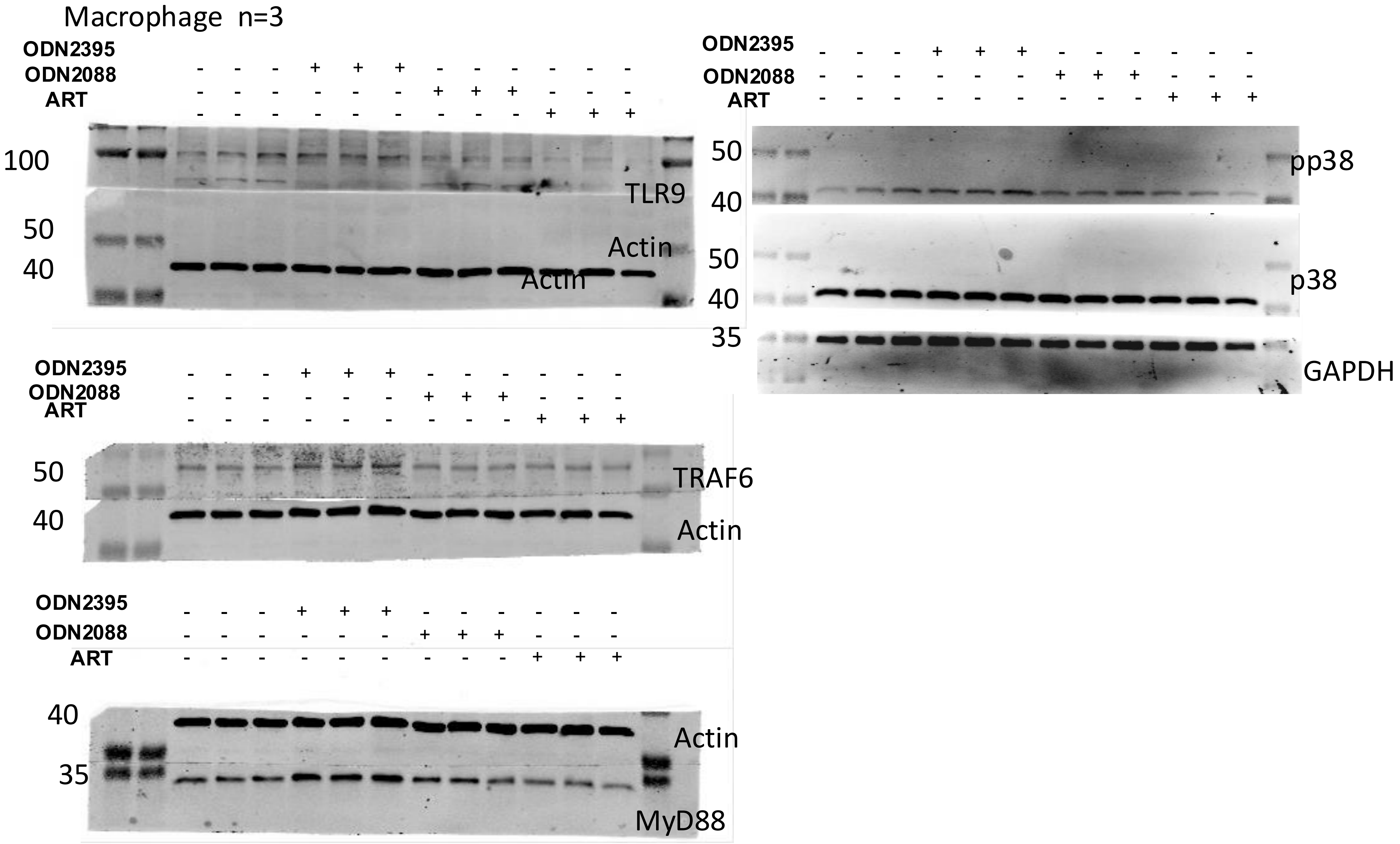
Macrophage n=3
ODN2395
ODN2088
ART
100
TLR9
50
40
Actin
Actin
ODN2395
ODN2088
ART
50
TRAF6
40
Actin
ODN2395
ODN2088
ART
40
Actin
35
MyD88
ODN2395
ODN2088
ART
50
40
pp38
50
40
p38
35
GAPDH

Figure S6

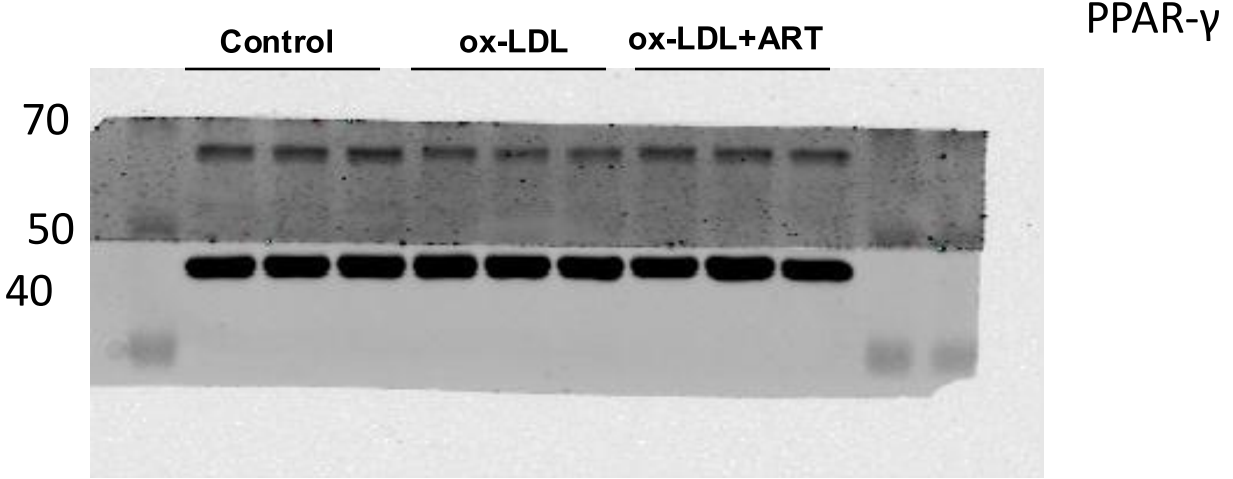

Figure S7

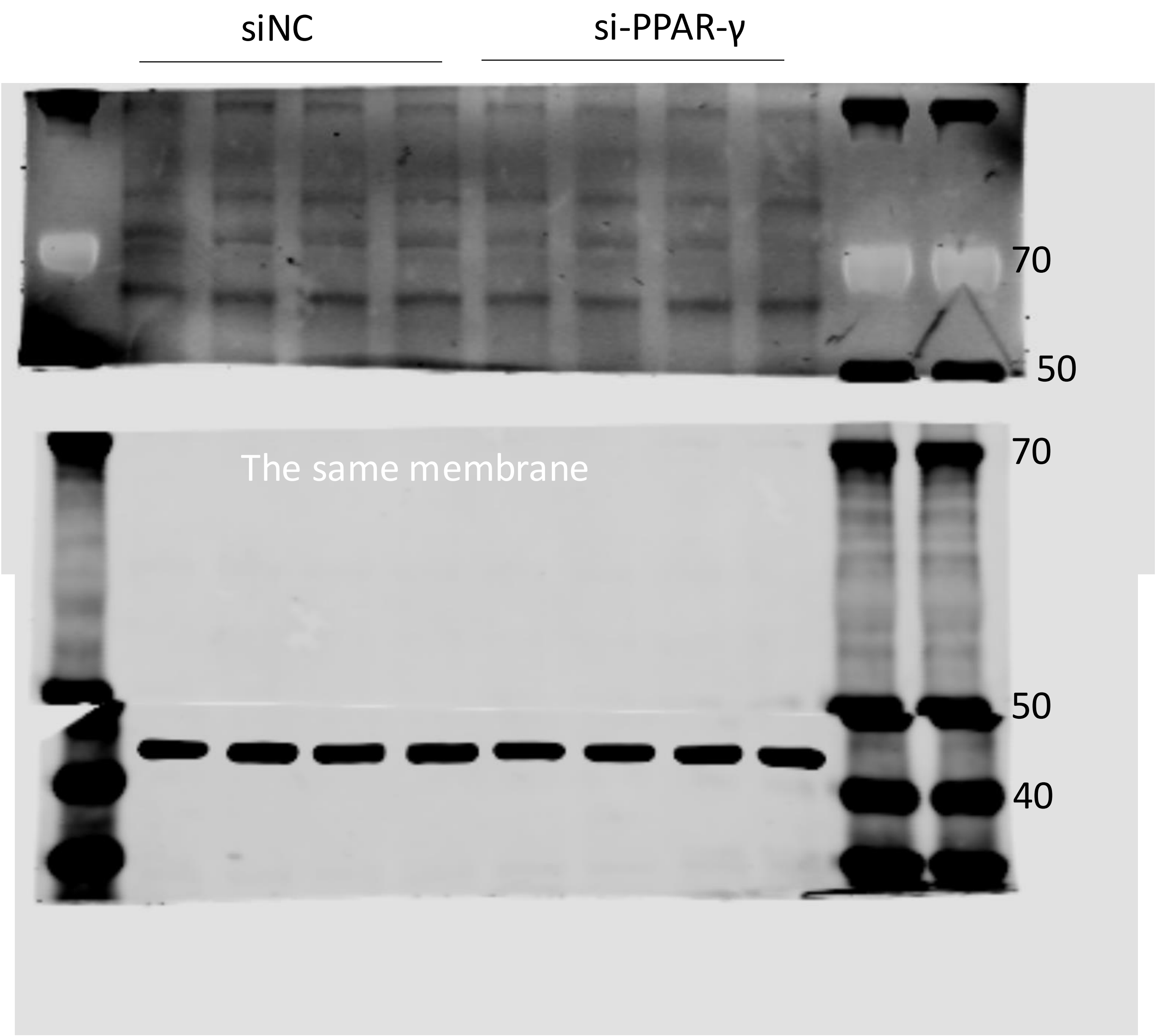

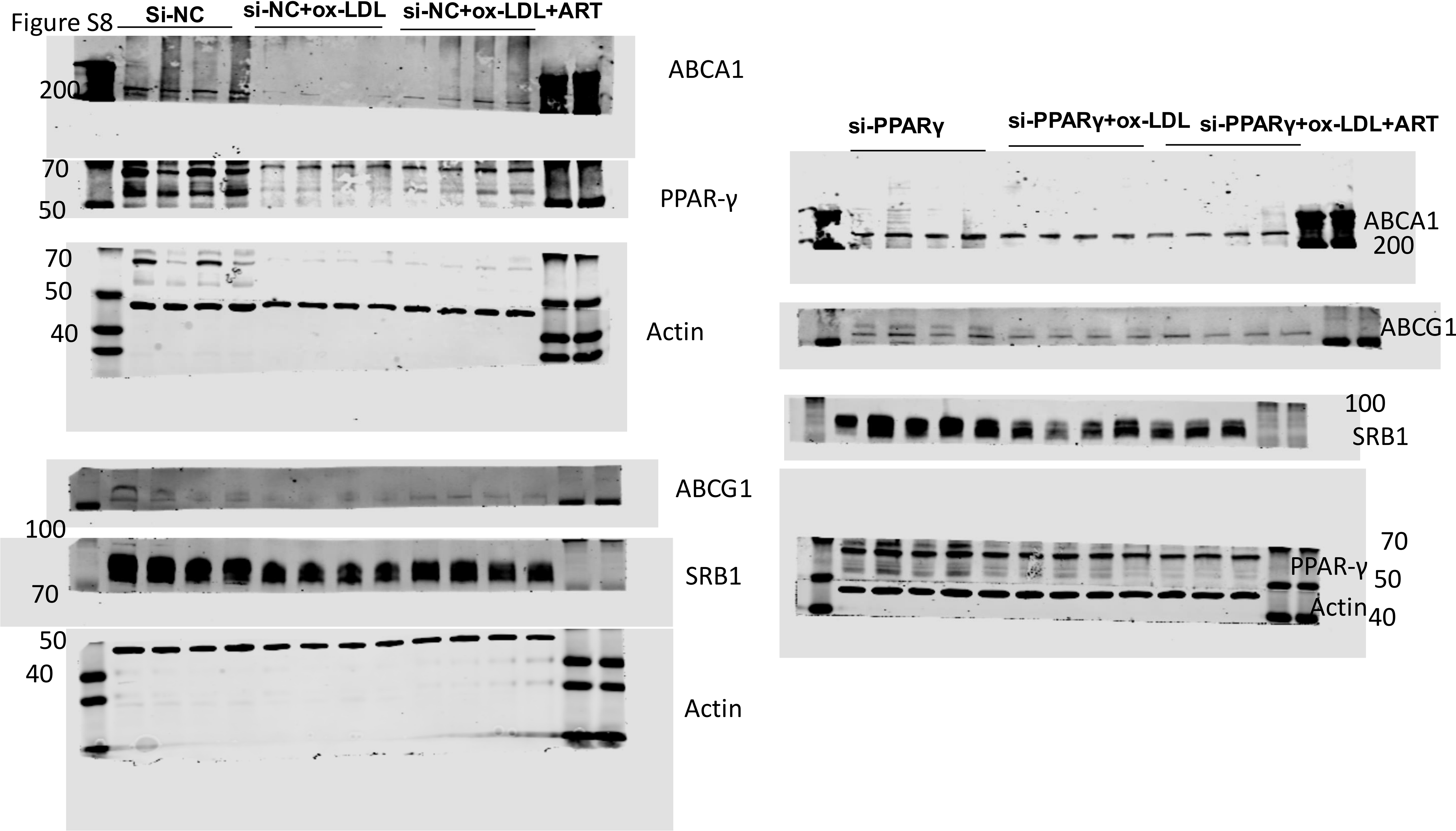
Si-NC
si-NC+ox-LDL
si-NC+ox-LDL+ART
ABCA1
200
70
50
PPAR-γ
70
50
40
Actin
ABCG1
100
SRB1
70
50
40
Actin
si-PPARγ
si-PPARγ+ox-LDL
si-PPARγ+ox-LDL+ART
ABCA1
200
ABCG1
100
SRB1
70
PPAR-γ
50
Actin
40

Figure S8
